# Parvovirus Infection Leading to Severe Anemia in an Adult Patient With HIV Disease

**DOI:** 10.7759/cureus.29148

**Published:** 2022-09-14

**Authors:** Myo Myint Tun, Tutul Chowdhury, Nway Nway, Pharlin Noel, Nicole Gousy, Aditi Roy, Shwe Yee Htet

**Affiliations:** 1 Internal Medicine, One Brooklyn Health System, Brooklyn, USA; 2 Surgery, Mount Sinai South Nassau Hospital, Oceanside, USA; 3 Medicine, American University of Antigua, New York, USA; 4 Internal Medicine, Sher-E-Bangla Medical College, Barishal, BGD

**Keywords:** immunocompromised, severe anemia, pure red cell aplasia, hiv infection, parvovirus b19

## Abstract

Individuals with human immunodeficiency virus (HIV) disease frequently suffer from anemia. The causes include anemia of chronic disease, vitamin B12 and iron deficiency, opportunistic infections (*Mycobacterium tuberculosis, Pneumocystis jiroveci*), HIV-related bone marrow suppression, AIDS-associated malignancies, and antiretroviral therapy (ART), specifically zidovudine. In HIV patients with advanced immunodeficiency, failure to produce neutralizing antibodies can lead to chronic parvovirus B19 (B19) infection. Normally, in persons with intact immunity, the progression of B19 is self-limited. However, in chronic B19 infection, it can lead to pure red cell aplasia (PRCA) and chronic anemia. In human immunodeficiency virus (HIV)-infected patients, B19-related anemia is rare and underdiagnosed. It has a great response to intravenous immunoglobulin (IVIG) therapy. Hence, early diagnosis and prompt treatment can significantly reduce mortality. In this article, we described the case of a 25-year-old male with HIV infection who presented with a headache. He had severe normocytic anemia with a low reticulocyte count. The workup for blood loss, hemolysis, hemoglobinopathy, and iron deficiency was negative. Because of extremely low reticulocytopenia with severe anemia, the investigations favored multiple myeloma, parvovirus infection, and bone marrow aspiration biopsy. He was tested for parvovirus B19 deoxyribonucleic acid (DNA) polymerase chain reaction (PCR) test due to insufficient seroconversion. It turned out to be positive and he was treated with IVIG therapy.

## Introduction

Parvovirus B19 (B19) was discovered in 1974 and is the only known pathogenic member of the family Parvoviridae [1]. B19 only infects humans and is transmitted primarily through respiratory droplets. However, it can also be transmitted through contaminated blood, organ transplantation, and vertical transmission [2]. Infection from B19 manifests differently depending on the immunologic and hematologic condition of the host [1]. B19 infection in a healthy host may result in self-limiting subclinical erythroid aplasia, followed by rash or arthralgia mediated by the immunological response. However, in immunocompromised hosts, chronic B19 infection manifests as pure red cell aplasia (PRCA) and chronic anemia [1]. In HIV-infected patients, reports of B19-related anemia are rare and probably underdiagnosed [3]. Persistent B19 infection is a clinically significant and curable cause of anemia in HIV-infected individuals [2,3]. Almost all B19-PRCA patients respond to intravenous immunoglobulin (IVIG) therapy with an increase in hemoglobin levels to adequate for the patient's clinical status [3]. It’s imperative to detect if an anemic HIV patient is infected with B19 in order to treat effectively [3] and choose antiretroviral (ART) medications carefully in order to avoid aggravating hematotoxic side effects such as severe aplastic anemia [2]. We present the rare case of a 25-year-old male with HIV infection who presented with severe normocytic anemia secondary to B19 infection. His only symptoms were occipital headache, dizziness, lethargy, palpitation, and decreased exercise tolerance. Workup for B19 was initiated. Our patient was treated accordingly and discharged from the hospital. We reviewed the literature and discussed B19 infection in HIV-infected patients, the clinical presentation, the management, and the therapeutic response of our patient.

## Case presentation

A 25-year-old male with a past medical history of HIV infection, anemia, hydrocephalus, and syphilis presented to the emergency department (ED) for sudden onset of persistent occipital headache for one month. The headache was described as dull aching pain with 10/10 in intensity. It has no triggering or aggravating factors. It was not associated with fever, confusion, nausea, vomiting, weakness, or urinary incontinence. He denied any prior episodes of this headache. A review of systems revealed lightheadedness, lethargy, palpitation, and reduced exercise tolerance for the same duration. He had visited three different emergency departments in the past few months for similar symptoms. In the last ED visit, he was found to have anemia with a hemoglobin of 3.3 mg/dl and a hematocrit of 9.9%. However, he refused further workup and signed out against medical advice. Family history revealed no blood-related malignancies. He was not compliant with his HIV treatment, Biktarvy.

On general assessment, he was alert but in mild distress due to a headache. Triage vitals were blood pressure of 110/52 mmHg, heart rate of 89/min, temperature of 37 °C (98.6 °F), and a respiratory rate of 16/min, with an oxygen saturation of 99% on room air. The physical exam was significant for the pallor of the conjunctiva, tongue, and palms. The neurological exam didn’t reveal any photophobia, neck stiffness, or gait disturbances. The CT scan of the head was negative for any lytic lesions. He was advised to have a lumbar puncture to rule out meningitis, but he refused. A complete blood count showed a hemoglobin of 2.7 mg/dl, a hematocrit of 7.8%, and a very low absolute retic count of 0.0011 × 10^6^/uL (Table 1). He received two units of an urgent packed red blood cell (pRBC) transfusion. Appropriate anemia workup was performed as per hematology consultation (Table 2).

**Table 1:** The complete blood count of the patient was taken during admission, revealing severe anemia. WBC: white blood cell count, HGB: hemoglobin, HCT: hematocrit, MCV: mean corpuscular volume, MCHC: mean corpuscular hemoglobin concentration, RDW: red cell distribution width, Retic Ct Abs: absolute reticulocyte count, and LD: lactate dehydrogenase.

Test	Ref range and units	Value
WBC	4.5-11.0 × 10^3^/uL	4.2 (L)
HGB	11.0-15.0 g/dL	2.7 (LL)
HCT	39-53%	7.8 (LL)
MCV	80-100 fL	80.5
MCH	26.0-33.0 pg	27.2
MCHC	30.5-36.0 g/dL	33.8
RDW	11.5-15.1%	14.6
Platelets	130-400 × 10^3^/uL	608 (H)
Reticulocyte count%	0.5-2%	0.11 (L)
Retic Ct Abs	0.024-0.09 × 10^6^/uL	0.0011 (L)
Bilirubin	0.2-1.2 mg/dL	0.3
LD	125-220 U/L	254 (H)

**Table 2:** Results of the patient’s complete blood cell count were taken at the time of presentation.

Test	Ref range and units	Value
Iron	49.0-181.0 ug/dL	274
Total iron binding capacity	240.00-450.00 ug/dL	334.03
Iron saturation	%	82
Ferritin	17.90-464.00 ng/mL	586.00 (H)
Transferrin	206.00-381.00 mg/dL	238.59
Cobalamin	232-1245 pg/mL	768
Folate	>3.0 ng/mL	11.10

A hemoglobinopathy evaluation showed a normal HGB A composition with no hemoglobin variant or thalassemia observed (Table 3).

**Table 3:** Results of the patient’s hemoglobinopathy evaluation ruling out any hemoglobin variant or thalassemia. HGB: hemoglobin.

Test	Ref range and units	Value
HGB F	0.0-2.0%	0.0
HGB A	96.4-98.8%	97.5
HGB A2	1.8-3.2%	2.5
HGB S	0.0%	0.0

The hemolysis workup with a direct Coombs test was negative. He had no prior episodes of bleeding and was also negative for occult blood loss in both urine and stool. Given reticulocytopenia in the background of severe anemia, further investigations were warranted for multiple myeloma, parvovirus infection, and bone marrow aspiration biopsy. The parvovirus deoxyribonucleic acid (DNA) polymerase chain reaction (PCR) test was a preferred test as seroconversion is delayed or insufficient, especially in HIV patients with an immunocompromised state (Tables 4, 5).

**Table 4:** This table shows the results of the hemolytic workup to gain further insight as to the cause of this patient’s severe anemia. UR: urinalysis; INR: international normalized ratio; and PTT: partial thromboplastin time.

Test	Ref range and units	Value
Blood, UA	Negative	Negative
Occult blood	Negative	Negative
Prothrombin time	9.8-13.4 sec	13.7
INR	0.85-1.15	1.12
PTT	24.9-35.9 sec	30.9
Direct Coombs	Negative	Negative

**Table 5:** This table shows the results of further investigation into his immunocompromised state with a positive parvovirus PCR result being of note. POS. Lymph: positive lymphocytes, PCR: polymerase chain reaction.

Test	Ref range and units	Value
Absolute CD4 helper	359-1519/uL	148 (L)
%CD4 POS. Lymph.	30.8-58.5%	5.6
Abs. CD8 suppressor	109-897/uL	1725
%CD8 POS. Lymph.	12.0-35.5%	61.6
CD4/CD8 ratio	0.92-3.72	0.09
Parvovirus B19, PCR	Negative	Positive

**Figure 1:**
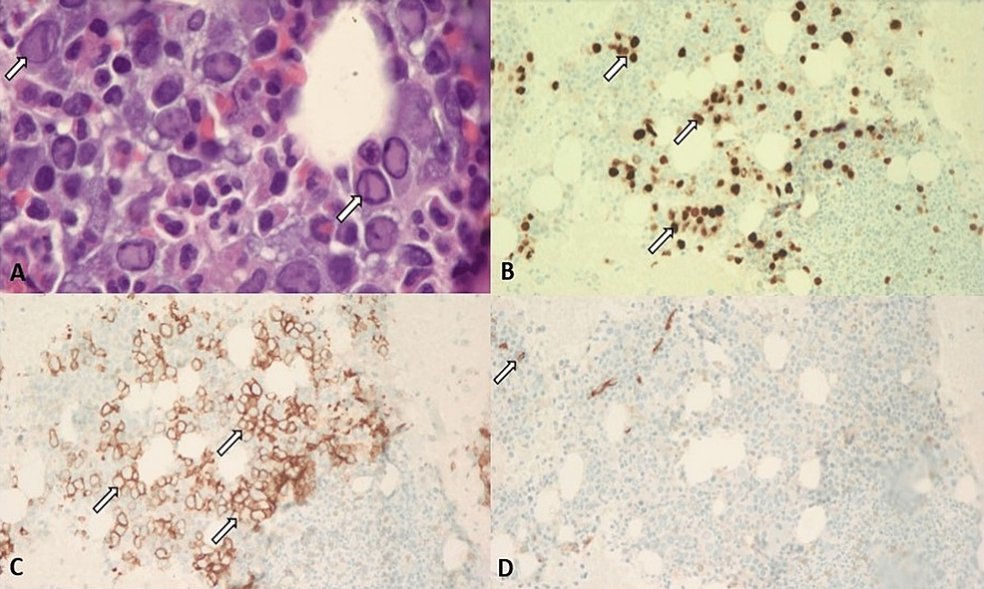
Results of the bone marrow biopsy. (A) H&E stain in bone marrow under 1000×: many cells with ground glass viral inclusions, consistent with parvovirus B19 infection (arrow). (B) IHC stain in bone marrow under 200×: parvovirus B19 (arrow). (C) E-cadherin IHC stain in bone marrow with 200×: highlighting erythroid precursor (arrow). (D) Bone marrow under 200×: CD34(+) blasts (arrow). IHC: immunohistochemistry.

The parvovirus PCR came back positive and, subsequently, he was treated with intravenous immunoglobulin (IVIG) at 1 g/kg for a total of 72.6 g per day. The infusion was given for two consecutive days. Further need for maintenance therapy was not recommended as absolute CD4 was more than 100/uL. The bone marrow aspiration biopsy result revealed scattered erythroid cells with ground glass viral inclusions. Immunohistochemistry stains confirmed the presence of many parvovirus B19-infected cells (Figure 1). There was no increase in blast counts, which rules out myeloproliferative disorders and leukemia. During his hospital stay, he received a total of five units of pRBC transfusion. The presenting symptoms eventually resolved with an improvement in reticulocyte count of 0.012 and stabilization in hemoglobin level (Hb/Hct: 7.8/22.7). He was discharged with extensive education and counseling about compliance with ART.

## Discussion

Parvovirus B19 (B19) is a deoxyribonucleic acid (DNA) virus that, among the Parvoviridae family, is the only member known to have a direct cytotoxic effect on human cells, specifically the erythroid progenitor cells [1]. B19 is transmitted either through vertical transmission or via respiratory droplets [2]. In children, infection with this viral pathogen results in a common childhood exanthem, the fifth disease, resulting in the pathognomonic “slapped cheek” rash [1]. In adults, however, the severity of the infection depends on the immunocompetency of the patient. In immunocompetent adults, infection with B19 can lead to general malaise and arthropathy. However, in immunocompromised patients, either from a pre-existing HIV infection or those on immunosuppressive regimens and patients with pre-existing hemolytic anemia, a B19 infection can result in an arrest of hematopoiesis with subsequent pure red cell aplasia (PRCA) [1]. In the patient we presented, his previous HIV infection with non-compliance ART led to an immunocompromised state making him vulnerable to the more severe consequences of B19 infection.

The resolution of infection of B19 relies solely on an intact and functioning immune system. An infection with B19 generally self-resolves with the production of specific antibodies that reduce viral action on erythroid progenitor cells by binding to the viral capsid [4]. As the adaptive immune system creates more IgM antibodies against B19, viremia declines and the subsequent destruction of erythroid progenitor cells declines accordingly [5]. B19-specific IgM and IgG antibodies are generally produced between two and three weeks, respectively, after primary B19 infection, and these antibodies are responsible for the elimination of B19 [6]. The symptoms of the fifth disease are a direct result of the formation of these IgM immune complexes [4].

However, in immunocompromised patients, there is a lack of IgM being produced to reduce the viral infectivity of B19, hence a sequela of prolonged destruction of erythroid progenitor cells is seen [3]. This may be explained in HIV patients due to the inability to perform basic antigen presentation by impaired or reduced numbers of macrophages or T-helper cells [4]. Since no immune complexes are being made, these patients routinely experience symptoms of anemia without other signs of infection. However, the diagnosis of anemia in those with HIV is rare, possibly due to underdiagnosing, or has been mistakenly attributed to the cytotoxic effects of ART [3,6]. In the rare cases of confirmed anemia in patients with HIV, there is still debate about whether the true cause of anemia is due to a B19 infection or if it is the effects of antiviral medication. Therefore, there must be a heightened suspicion for B19 in an immunocompromised patient presenting with sudden-onset signs of anemia, such as seen in the patient presented here.

In those with HIV, the B19 virus can remain undetected for prolonged periods of time. The virus has the ability to stay within a cellular reservoir, resulting in relapsing and remitting bouts of a B19 infection [3,4,6,7]. This can be combated by the use of intravenous administration of IgG antibodies against the B19 viral capsid [5-7]. It is increasingly important for patients with HIV to be persistently monitored for persistent B19 infection. A crucial caveat to keep in mind with monitoring for repeating relapses of B19 is the use of zidovudine within the ART protocol. It has been noted that the use of zidovudine can complicate reticulocyte counts due to its propensity for bone marrow suppression. This can be overcome by determining the serum B19 DNA levels in a patient with HIV [4,8]. Studies have shown that repeated administration of IVIG can be especially helpful in treating red cell aplasia in those with chronic B19 to reduce erythroid progenitor cell destruction [4,8].

## Conclusions

Given its rare and unique course of disease depending on immunologic status, the presence of B19-PRCA reflects the severity of immunodeficiency in ART non-adherence HIV patients. In fact, we may consider B19-PRCA to be an opportunistic infection with advanced immunodeficiency. It seems to be the case with our patient who is not adherent to ART with low CD4 counts. Therefore, a high index of suspicion for B19-related anemia should be concerned for HIV patients with severe and long-standing anemia. Even though IVIG therapy is the mainstay of treatment to clear viremia, we have to be aware that compliance with ART for early reconstitution of immunity is also essential to prevent relapse of B19 and resolution of anemia.
